# Retrotransposons Down- and Up-Regulation in Aging Somatic Tissues

**DOI:** 10.3390/cells11010079

**Published:** 2021-12-28

**Authors:** Giorgia Giordani, Valeria Cavaliere, Giuseppe Gargiulo, Giovanna Lattanzi, Davide Andrenacci

**Affiliations:** 1 Dipartimento di Farmacia e Biotecnologie, Alma Mater Studiorum Università di Bologna, 40126 Bologna, Italy; giorgia.giordani3@unibo.it (G.G.); valeria.cavaliere@unibo.it (V.C.); giuseppe.gargiulo@unibo.it (G.G.); 2 CNR Institute of Molecular Genetics “Luigi-Luca Cavalli-Sforza” Unit of Bologna, 40136 Bologna, Italy; giovanna.lattanzi@cnr.it; 3 IRCCS Istituto Ortopedico Rizzoli, 40136 Bologna, Italy

**Keywords:** longevity, transposons, *gypsy*, *ZAM*, *Idefix*

## Abstract

The transposon theory of aging hypothesizes the activation of transposable elements (TEs) in somatic tissues with age, leading to a shortening of the lifespan. It is thought that TE activation in aging produces an increase in DNA double-strand breaks, contributing to genome instability and promoting the activation of inflammatory responses. To investigate how TE regulation changes in somatic tissues during aging, we analyzed the expression of some TEs, as well as a source of small RNAs that specifically silence the analyzed TEs; the *Drosophila* cluster named *flamenco*. We found significant variations in the expression levels of all the analyzed TEs during aging, with a trend toward reduction in middle-aged adults and reactivation in older individuals that suggests dynamic regulation during the lifespan.

## 1. Introduction

Transposable elements (TEs) are DNA sequences able to proliferate in genomes by mechanisms that allow these elements to transpose into new sites. Among TEs, retrotransposons are characterized by a mechanism of replication through reverse transcription of an RNA intermediate. Retrotransposons are, in turn, classified as either long terminal repeats (LTR) retrotransposons or non-LTR retrotransposons. In *Drosophila*, a specific group of retrotransposons are repressed in the somatic cells of adult ovaries by the activity of a small RNA cluster named *flamenco* [1]. Like other small RNA clusters, *flamenco* is constituted of TE fragments, which are expressed in the form of a precursor long non-coding RNA. The transcript is then processed to produce Piwi-interacting RNAs (piRNAs) that silence the regulated TEs. In somatic tissues outside the ovary, endogenous small interfering RNAs (endo-siRNAs) mapping to the *flamenco* locus were also found [2], suggesting its involvement in the regulation of TEs outside the reproductive organs, as well. Among the mobile elements controlled by *flamenco* there are at least three LTR-retrotransposons: *gypsy*, *ZAM* and *Idefix* [3,4,5]. It is known that the ability to silence these mobile elements is variable and there are *Drosophila* lines that are permissive for their mobilization and lines that are non-permissive [3,5]. A well-characterized line permissive for the mobilization of ZAM and Idefix (Rev line) displays large deletions that remove *ZAM* and *Idefix* sequences in the *flam*enco locus [6]. This allows *ZAM* and *Idefix* expression in the follicular epithelium and in somatic tissues outside the ovary throughout *Drosophila* development (in embryos, larvae and adult flies) [7]. Permissive lines for *gypsy* mobilization have also been characterized, allowing *gypsy* activation in follicular cells and in the head tissues [8,9,10]. Regulation of TEs by *flamenco* involves the PIWI protein in the reproductive apparatus, and is PIWI independent in the somatic tissues, suggesting different molecular mechanisms of repression [7]. Although these mechanisms have been investigated, nothing is known about what happens to the expression of small RNA clusters in aging, and whether there is a correlation with changes in the expression levels of the regulated TEs. Increased mobilization of some TEs in somatic tissues during aging has been considered to be a cause of lifespan-shortening, and the observation of this phenomenon is at the base of the “transposon theory of aging” [11,12]. Reduction of constitutive heterochromatin during lifespan [13] is considered to be the cause of the derepression of a number of TEs, which are mainly located in heterochromatin regions [14]. Their transcriptional activation, which is associated to an increase of transposition events, increases the production of DNA double-strand breaks (DSBs), contributing to genome instability and the activation of inflammatory responses [15,16,17].

Considering the role of the small RNA clusters in TE silencing, we wondered what happens to the expression of *flamenco* and some TEs that are potentially regulated by this cluster in aging, using allele combinations that produce flies able or unable to repress *gypsy*. Analyses were performed comparing these expressions in the head tissues of young, middle-aged and old females. We found a substantial repression of most of the analyzed sequences in middle-aged flies, and increased expression in old flies. Expression of retrotransposons and *flamenco* were different when comparing young flies able to repress *gypsy* with those unable to repress *gypsy*, while they were very similar in old flies. This suggests differing capabilities to repress TE at different points during the lifespan. Furthermore, flies with a higher level of *gypsy* expression showed a small reduction in longevity with respect to individuals demonstrating lower *gypsy* expression. This finding supports a possible correlation between *gypsy* activation and longevity.

## 2. Materials and Methods

### 2.1. Drosophila Stocks

*Drosophila* stocks were kept at 25 °C on a standard cornmeal/yeast medium. Old flies were collected for each genotype and used for the experiments. To obtain middle-aged and old flies, young individuals were collected in freshly prepared food vials that were kept horizontally. Food vials were changed every three days. The following strains were used: *Canton S* (wild type), *flam^A^*, *w^IR6RevII7^*, *Df(1)l11/FM7c B flam^FM7^; P{gypsy-lacZ.p12}* [8] and *flam^BG02658^*. *Df(1)l11* deletion includes *eor*, *flam*, and *wap* genes [18]. Since the non-permissive flies carry the *Bar* (*B*) dominant mutation, these flies were distinguished from the permissive ones on the base of the dominant Bar phenotype of the eyes.

### 2.2. Quantitative RT-PCR

Total RNA was extracted by crushing in TRI Reagent (Sigma-Aldrich, St. Louis, MO, USA) five ovaries (three days old), or ten female heads of different selected ages. The removal of genomic DNA contamination and synthesis of the complementary cDNA was performed by HiScript III 1st Strand cDNA Synthesis Kit (+gDNA wiper) (Vazyme, Nanjing, China), according to the manufacturer’s protocol. Gene expression analysis was performed using the Power SYBR Green PCR master mix (Thermo Fisher Scientific, Waltham, MA, USA) in 96-well Piko PCR Plates (Thermo Fisher Scientific, Waltham, MA, USA) using the PikoRel 96 Real-Time PCR System (Thermo Fisher Scientific, Waltham, MA, USA), according to the manufacturer’s suggested procedure. To determine target specificity, dissociation curve analysis was performed. RNA levels were normalized to the internal standard gene *Rp49*. The ΔΔCt method was used to measure the expression levels. The calibrator used for each experiment is reported in the figure legend. Three biological replicates were used for each experiment, and the average was calculated. All reported errors are the standard deviation of the mean. For quantitative PCR (qPCR) primers see Supplemental Material, Table S1 [19].

### 2.3. Lifespan Analysis

Mated females were used in the experiments. Animals were maintained in the climate chamber at 25 °C on a standard cornmeal/yeast medium in a 12 h light, 12 h dark regime. Three *Drosophila* vials containing 20 females per vial were used for each genotype. Animals were relocated to a fresh medium every three days. Dead flies were counted daily.

### 2.4. Statistical Analyses

For the comparison of two groups with the same age and different genotype, P-values were calculated using an unpaired *t*-test. Where multiple genotypes were compared, a one-way ANOVA was performed, applying a Tukey post hoc test. Where multiple ages and genotypes were compared, a two-way ANOVA was performed, and the results are reported in the figure legends. For the comparison of two groups with different ages, a post hoc analysis was performed, applying the LSD test.

Survival analyses for the lifespan curves were performed using the Kaplan-Meier method with the Log Rank test. The calculation of the average lifespan was performed with this formula: (day 1 × dead numbers of this day + day 2 × dead numbers of this day + …day n × dead numbers of this day)/total dead numbers. Standard deviation was also calculated.

IBM SPSS software version 25 was used for all of the statistical analyses.

## 3. Results

### 3.1. Gypsy, ZAM and Idefix Expression Is under Flamenco Regulation in Head Tissues

**Figure 1:**
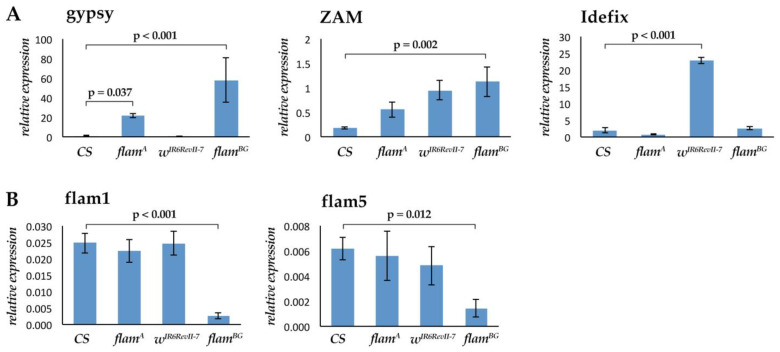
*gypsy*, *ZAM* and *Idefix* retrotransposons are under the regulation of *flamenco* in head somatic tissues. (**A**,**B**) Gene expression analysis in RNAs isolated from female head tissues from *Canton S* (*CS*), *flam^A^ /flam^A^*, *w^IR6RevII7^/w^IR6RevII7^* and *flam^BG^ /flam^BG^* strains. The fold changes indicate the expression levels relative to calibrator (gypsy in *CS*). (**A**) Expression analysis of *gypsy*, *ZAM* and *Idefix*. (**B**) Expression analysis of *flamenco*. Shown are average levels (*n* = 3), and error bars indicate SD. Significance was determined by one-way ANOVA with Tukey post hoc test.

We analyzed the expression of *gypsy*, *ZAM* and *Idefix*, comparing the *Canton S* (*CS*) wild type strain with the *flam^A^* strain, which carries a *flamenco* permissive allele for *gypsy* mobilization [19], and the *w^IR6RevII7^* strain, which carries a *flamenco* permissive allele for *Idefix* mobilization [20,21]. The *CS* strain showed low expression levels for all three analyzed retrotransposons (Figure 1A). On the other hand, the *flam^A^* strain showed a high *gypsy* expression level and low *ZAM* and *Idefix* levels, while *w^IR6RevII7^* strain showed a high *Idefix* expression level and low *gypsy* and *ZAM* expression levels (Figure 1A). These data confirm that *gypsy* and *Idefix* are upregulated in the head when there is a specific permissive allele of *flamenco*, similarly to what happens in ovaries. Using a couple of primers designed to amplify the 5′ coding region of *flamenco* (flam1) (Figure S1), we found very similar transcriptional levels of *flamenco* in the three genetic backgrounds (Figure 1B). Similar results were obtained using a second couple of primers (flam5) designed downstream of flam1 (Figure 1B and Figure S1). To further support the hypothesis that *flamenco* is involved in the regulation of the analyzed retrotransposons in the head’s somatic tissues, we analyzed the effect of the *flam^BG^* allele. It is reported that this allele, which derives from the P element insertional mutation in the 5′ of the *flamenco* locus (Figure S1), is permissive for *gypsy* and *ZAM* expression in the follicular epithelium [4]. RT-qPCR experiments have confirmed that *gypsy* is strongly upregulated, while *flamenco* is downregulated in the ovary [1]. Expression analysis in *flam^BG^* head tissues confirmed that *gypsy* and *ZAM* are significantly upregulated compared to *CS* (Figure 1A), while *flamenco* is significantly downregulated (Figure 1B). All these data are in agreement with the hypothesis that *flamenco* has a role in the regulation of the three retrotransposons in the head tissue.

### 3.2. Aging Effects on the Expression of Flamenco and Flamenco Regulated Retrotransposons

**Figure 2:**
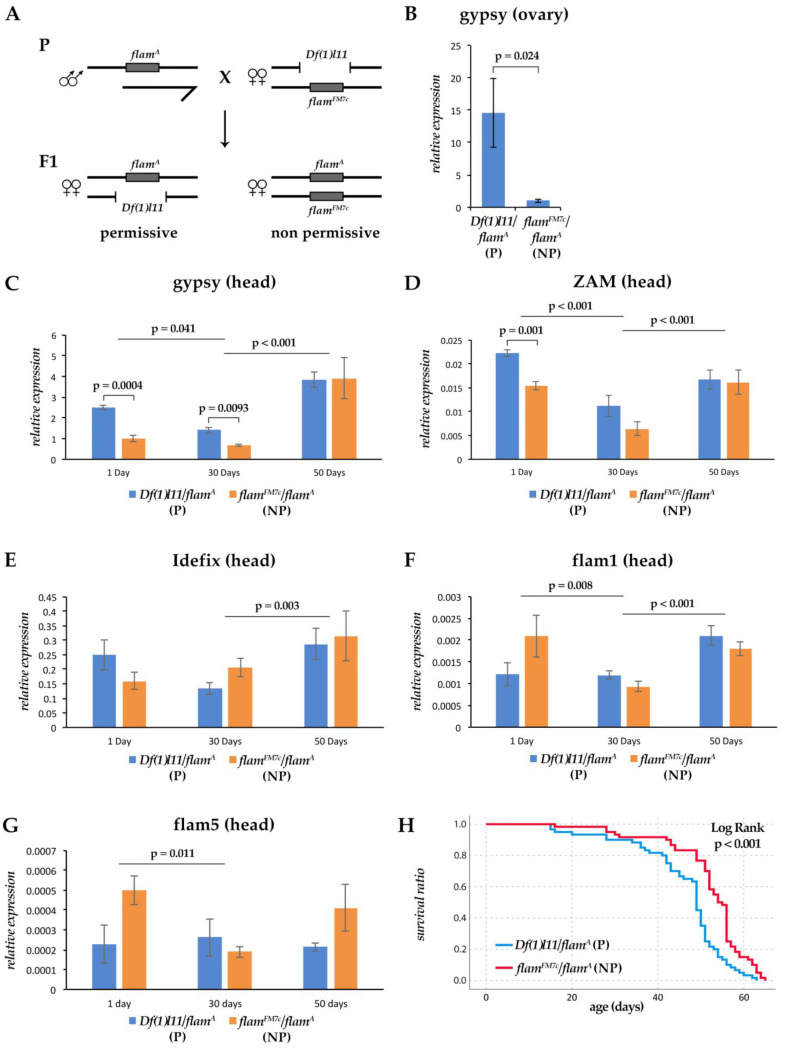
Analysis of the expression of the selected retrotransposons and of *flamenco* in aging. (**A**) Schematic representation of the genetic cross performed to obtain individuals permissive and non-permissive for the activation of *gypsy*. (**B**) Analysis of the expression of *gypsy* in RNAs isolated from 3-days-old ovaries of *flam^A^/Df(1)l11* and *flam^A^*/flam^FM7^ adult females. The fold changes indicate the expression levels relative to calibrator (*flam^A^*/*flam^FM7^*) (**C**–**F**) Gene expression analysis in RNAs isolated from head tissues from *flam^A^/Df(1)l11* and *flam^A^*/*flam^FM7^* adult females on Day 1 (young), Day 30 (middle-aged) and Day 50 (old). The fold changes indicate the expression levels relative to calibrator (gypsy in 1 day-old *flam^A^*/*flam^FM7^*) (**C**) gypsy expression. A two-way ANOVA reveals a significant effect of genotype (*p* = 0.015) and of age (*p* = 0.001). (**D**) ZAM expression. A two-way ANOVA reveals a significant effect of genotype (*p* = 0.002) and of age (*p* = 0.001). (**E**) Idefix expression. A two-way ANOVA reveals a significant effect of age (*p* = 0.009). (**F**) flam1 expression. A two-way ANOVA reveals a significant effect of genotype/age (*p* = 0.011) and of age (*p* = 0.001). (**G**) flam5 expression. A two-way ANOVA reveals a significant effect of genotype/age (*p* = 0.007), of genotype (*p* = 0.004) and of age (*p* = 0.034). Shown are average levels (*n* = 3), and error bars indicate SD. Comparison of two groups with different ages was done by applying a LSD post hoc analysis. Pairwise comparisons between genotypes of the same age were done using a two tails unpaired *t*-test. (**H**) Kaplan-Meier survival curves of *Df(1)l11/flam^A^* and *flam^FM7C^/flam^A^* adult females.

We decided to analyze the expression of these three TEs and of *flamenco* in female heads during aging. Analyses were performed on Day 1 (young), Day 30 (middle-aged) and Day 50 (old) flies. We also tested whether a different ability to silence at least one retrotransposon could affect the regulation of TEs in aging. To this end, we analyzed the expression of the TEs and *flamenco* in two groups of flies derived from the same genetic cross and carrying a different *flamenco* allelic combination in a similar genetic background (Figure 2A). We used the *flam^A^* strain because the high *gypsy* expression level found in this strain is associated to high retrotransposon activity [19]. To obtain flies that were non-permissive (NP) and permissive (P) for *gypsy* mobilization, we crossed *flam^A^* males (P) with *Df(1)l11/flam^FM7^* females [19]. *Df(1)l11* deletion encompasses the *flamenco* locus, while the *flam^FM7^* is a dominant *gypsy* non-permissive allele carried by an *FM7c* balancer (Figure 2A). From this cross, *flam^A^/Df(1)l11* (P) flies were obtained, which carried a *gypsy* permissive allele in the hemizygous condition, and *flam^A^/flam^FM7^* (NP) flies, with a permissive and a dominant non-permissive allele. As expected, we found that, in adult ovaries, *gypsy* expression was significantly higher in P than in NP flies (Figure 2B). A significantly higher expression level of *gypsy* in P flies was also found in the head tissues of young adult females (Figure 2C). These data confirm that *flamenco* is involved in *gypsy* regulation in the head, even if the effect of *flamenco* on the regulation of *gypsy* in these somatic tissues seems to have a minor impact. Comparing the expression of the three retrotransposons regulated by *flamenco* in young individuals of the two genetic backgrounds, we found that all showed slightly lower expression levels in the NP genetic background (Figure 2C–E). Furthermore, *flamenco* expression was two times higher in NP versus P flies (Figure 2F) due to the presence of a single copy of the *flamenco* gene in the latter.

We then analyzed variations in the expression levels of the three TEs and of *flamenco* in aging. We found that the significant difference in the expression levels of *gypsy* between P and NP was maintained in middle-aged flies (Figure 2C). A significant reduction in the expression levels was observed for *gypsy*, *ZAM* and *flamenco* in the transition from young to middle-aged flies, while *Idefix* shows an opposite pattern for P and NP (Figure 2C–G). In the transition from middle-aged to old flies, the expression levels of all the analyzed TEs were significantly upregulated, becoming quite similar in P and NP flies (Figure 2C–E). A similar activation effect was also observed for *flamenco*, even if small differences were observed when comparing the expression of the two regions analyzed (Figure 2F,G). These data show that the differences in the expression levels of *gypsy* and *ZAM* found at Day 1 and Day 30 between P and NP disappear in old individuals.

We found longevity differences between P and NP flies, which were manifested after the first 30 days of life, with P flies showing a slightly higher mortality rate (Figure 2H). The average lifespan of P flies was 46.22 ± 1.39 days while that of NP flies was 52.25 ± 1.2 days.

## 4. Discussion

The reported data suggest that the expression of TEs can change with a down- and up-regulation trend during aging. In fact, after the first half of the *Drosophila* adult life (Day 30), we found a significant down-regulation for two of the retrotransposons analyzed (*gypsy* and *ZAM*) in both the genetic backgrounds and for *Idefix* in the P genetic background (Figure 2). In the transition from middle-aged to old flies (from Day 30 to Day 50), we found a significant increase in the expression levels of all of the retrotransposons analyzed. Retrotransposon down- and up-regulation has been also described for the *Drosophila Copia* element in adult testis, in which it is down regulated in 12–15-day-old tissues with respect to three-day-old tissues and again upregulated at 24–27 days [22].

**Figure 3:**
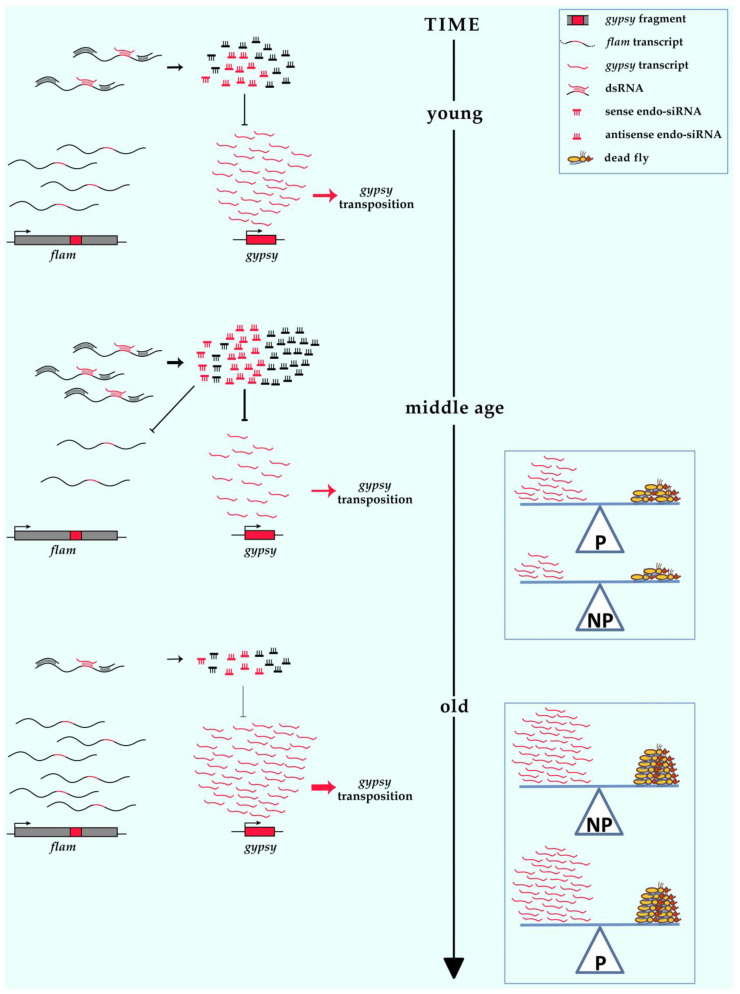
A speculative model for *gypsy* regulation during the aging process. *flamenco* is highly enriched in antisense TE sequences, comprising *gypsy* fragments. The *flamenco* primary transcript can form double strand RNAs (dsRNAs) with *gypsy* sense transcripts that serve as substrates for the production of endo-siRNAs specific for the silencing of *gypsy*. In young flies, *gypsy* expression level depends on its transcriptional rate and on post-transcriptional silencing induced by specific endo-siRNAs (*gypsy*-antisense). In middle age, reduction in *gypsy* and *flamenco* expression can be explained by an increased production of endo-siRNAs (sense and antisense) specific for the silencing of *gypsy* and *gypsy* fragments that are part of the *flamenco* transcript (cosuppression). In old flies, *gypsy* and *flamenco* appeared derepressed, and this can be explained by a reduction of silencing, probably due to a reduction in the amount of endo-siRNAs (sense and antisense). Similar mechanisms can be hypothesized to explain down- and up- regulation of *ZAM* and *Idefix* in aging. Right boxes: a legend is represented in the upper box; correlation between the abundance of *gypsy* transcription and mortality in P(permissive) and NP (non-permissive) flies in middle aged individuals (middle age box) and old individuals (old box).

The enhanced ability of NP somatic tissues to repress *gypsy* and *ZAM* expression in young individuals is no longer found in old individuals. Aging seems to smooth out differences in the regulation of retrotransposons between P and NP somatic tissues. The expression of *flamenco*, one of the main regulators of these TEs, changes over time in a similar way to that of the retrotransposons under its control. The significant reduction levels of transcript in middle-aged individuals could depend on mechanisms of cosuppression, as previously described for *gypsy* and *flamenco* [19], which could explain the concomitant repression of *gypsy*, *ZAM* and *flamenco*. Cosuppression, which has been described to act at the post transcriptional level [19], is triggered by *gypsy* proliferation as a consequence of the high retrotransposon expression level (Figure 3). This mechanism suppresses the deleterious effect of *gypsy* proliferation, which would inevitably lead to higher expression levels and higher transposition rates. Endo-siRNAs, sense and antisense to TE sequences, have been found in somatic tissues [2], and their increase could explain the increased silencing of both retrotransposons and *flamenco*. Activation in old age of both retrotransposons and *flamenco* could be explained by the accumulation of unprocessed *flamenco* transcripts, and a reduction in the production of small RNAs that serve to silence the target TEs (Figure 3). Furthermore, high expression levels could also be sustained by the derepression of retrotransposon copies that could be located in heterochromatin regions, in response to the reduction of constitutive heterochromatin that is found in old individuals [14].

The difference in the average lifespan between *gypsy* permissive and non-permissive flies (Figure 2H) supports the idea that *gypsy* activation could be involved in the shortening of lifespan (Figure 3). Significantly, a shorter lifespan has been described in *Ago2* mutations, which disrupt the somatic TE control mechanism and increase expression of the retrotransposons *gypsy* and *R2* in brain [23]. It has also been described that lines exhibiting earlier *gypsy* transposition events showed a shorter lifespan, while those with later transposition events showed a longer lifespan [17]. All of these results are in agreement with the hypothesis of the involvement of TE activation in reducing the lifespan.

As a whole, our study suggests that the investigation of a possible role for the distribution of TEs in the genome and of their activation state during aging could shed light on physiological and pathogenetic differences among aging individuals. The study of the role of small RNA clusters in regulating TE expression in aging could help to identify the causes of expression variation of some TEs in aging.

## Data Availability

Not applicable.

## Supplementary Materials

The following are available online at https://www.mdpi.com/article/10.3390/cells11010079/s1, Figure S1: Schematic representation of the *flamenco* locus and the regions where the specific primer pairs map; Table S1: Primer sequences used in this study.
