# Supplementary material for: Retrotransposons Down- and Up-Regulation in Aging Somatic Tissues
Source: Cells. 2021 Dec 28;11(1):79. doi: 10.3390/cells11010079 (PMC8750722; doi:10.3390/cells11010079)
Supplement: Supplementary file 1 [file cells-11-00079-s001.zip › cells-1470911-supplementary.pdf]

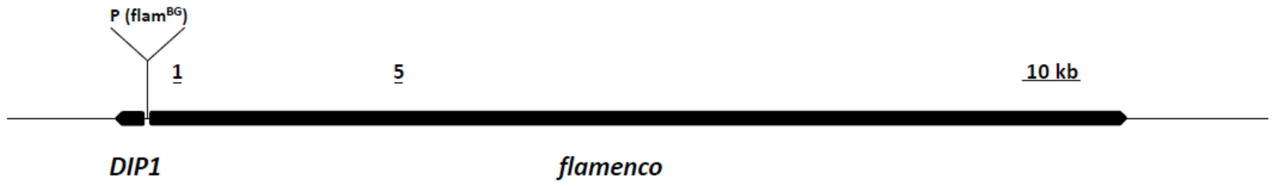

Figure S1. Schematic representation of the *flamenco* locus and the regions where the specific primer pairs map. Map of the *flamenco* gene showing the *DIP1* gene, the *flam*^{BG02658} (flam^{BG}) insertion at the 5′ end of the *flamenco* locus, and the positions of primers used to analyze *flamenco* primary transcripts expression (1-flam1;5-flam5).

Table S1. Primer sequences used in this study.

Primers for qPCR	Forward Primer	Reverse Primer
rp49	5’ TCTGCATGAGCAGGACCTC 3 ’	5’ ATCGGTTACGGATCGAACAA 3 ’
gypsy	5’ AGACGCTGCGACCATTCAC 3’	5’ CGTGCTGCCTCCAGAATGAT 3’
flam1	5’ TCAAAGCGATTCATTCCTCAG 3’	5’ CCATTTGGCTATGAGGATCAG 3’
flam5	5’ CAGGCCCCCTATTGATTAGAT 3’	5’ TGCTCGGGCTTTCTTAAAGT 3’
ZAM	5’ TTCGCGTTAGGAGCCGTACT 3’	5’ TTGACTTCGGTGTCGGAGAG 3’
Idefix	5’ GAATGATTCCGCTCTAGTGG 3’	5’ ATGCGGTCTCTTTCTTCTGC 3’
